# A Novel *DPYD* Variant Associated With Severe Toxicity of Fluoropyrimidines: Role of Pre-emptive *DPYD* Genotype Screening

**DOI:** 10.3389/fonc.2018.00279

**Published:** 2018-07-24

**Authors:** Chi C. Tong, Ching W. Lam, Ka O. Lam, Victor H. F. Lee, Mai-Yee Luk

**Affiliations:** ^1^Department of Clinical Oncology, Queen Mary Hospital, Hong Kong, Hong Kong; ^2^Department of Pathology, University of Hong Kong, Pokfulam, Hong Kong; ^3^Department of Clinical Oncology, University of Hong Kong, Pokfulam, Hong Kong

**Keywords:** novel, *DPYD* variant, fluoropyrimidines, pharmacogenetics, precision medicine

## Abstract

**Background:** The fluoropyrimidine anticancer drug, especially 5- fluorouracil (5-FU) and its prodrug capecitabine are still being the backbone of chemotherapeutic regimens for colorectal cancer. Dihydropyrimidine dehydrogenase (DPD) is the crucial enzyme in the catabolism of 5-FU. Over the past 30 years, there is substantial clinical evidence showing that DPD deficiency is strongly associated with severe and fatal fluoropyrimidine-induced toxicity.

**Patients and methods:** A 49-year-old lady with resected stage III carcinoma of sigmoid colon was scheduled to have a course of 5-FU based adjuvant chemotherapy. She developed unexpected acute severe (grade 4) toxicity after the first cycle of chemotherapy. Genomic DNA was isolated from 3 ml peripheral blood cells for full sequencing of ***DPYD*** (the gene encoding DPD).

**Results:** Exome sequencing confirmed that she is heterozygous for NM_000110.3: c.321+2T>C of the ***DPYD*** gene. To the best of our knowledge, this variant is a novel pathogenic splicing variant of the ***DPYD*** gene resulting in a non-functional allele. As she has a heterozygous genotype and considered having decreased DPD activity, we followed the international recommendation and restart chemotherapy with at least 50% reduction for 5-FU dose. We then titrated the 5-FU dose, and she tolerated the subsequent cycles of chemotherapy and completed the whole course of adjuvant chemotherapy.

**Conclusions:** With a pre-emptive test on DPD deficiency before the administration of the fluoropyrimidine drugs, the aforementioned patient's life-threatening event could be avoided. This clinical utility has been confirmed by two recent large-scale studies and called for a drug label update.

## Introduction

Dihydropyrimidine dehydrogenase (DPD) plays a key role in the catabolism of 5- fluorouracil (5-FU). The ***DPYD*** gene encodes this enzyme. There is substantial evidence showing genetic polymorphism of ***DPYD*** is the leading cause of deficiency in enzymatic activity, resulting in DPD deficiency syndrome and manifest excessive toxicity including severe diarrhea, mucositis, and pancytopenia after treatment with fluoropyrimidines (1).

Here we present a case report about a patient with a novel mutation in the ***DPYD*** associated with severe toxicity of fluoropyrimidines. The role of pre-emptive DPD genotype screening to improve the efficacy versus toxicity ratio in the era of precision medicine is also discussed.

### Case report

A 49-year-old lady was diagnosed to have carcinoma of sigmoid colon with laparoscopic left hemicolectomy done in early September 2016. Pathology revealed moderately differentiated adenocarcinoma; tumor involved per-colic tissue, one out of 14 regional lymph nodes was positive, all resection margins were clear, hence pathologically staged T3N1M0, stage III disease.

She was planned to have a course of adjuvant chemotherapy with CAPEOX [capecitabine (1000 mg/m2 twice daily oral for 14 days) and oxaliplatin (130 mg/m2 at day 1)] for eight cycles, with an aim to achieve 10% absolute gain in 8-year overall survival (2). The first cycle of CAPEOX was started in late October 2016.

At Day 13, she developed fever and grade 3–4 diarrhea (CTCAE v 4.0) (3) and day 14 capecitabine being withheld. She was then admitted into the hospital through Emergency Department. Complete blood count revealed that she had grade 4 marrow suppression toxicity: total white blood cell: 0.52 × 10^∧^9/L (normal range: 3-89–9.93 × 10^∧^9/L), neutrophil: 0.04 × 10^∧^9/L (normal range: 2.01–7.42 × 10^∧^9/L), platelet: 25 × 10^∧^9/L (normal range: 154–371 × 10^∧^9/L). She necessitated intravenous antibiotics with piperacillin/tazobactam and growth factors were given for neutropenic fever. She was discharged home after hospitalization care for 3 weeks.

In view of stormy side effects of chemotherapy, she was clinically highly suspected to have DPD deficiency.

## Methods

The patient signed a written informed consent for genotyping and the data being used for scientific publication. The targeted exome analysis for ***DPYD*** genotypes was performed as described previously (4).

## Results

**Figure 1:**
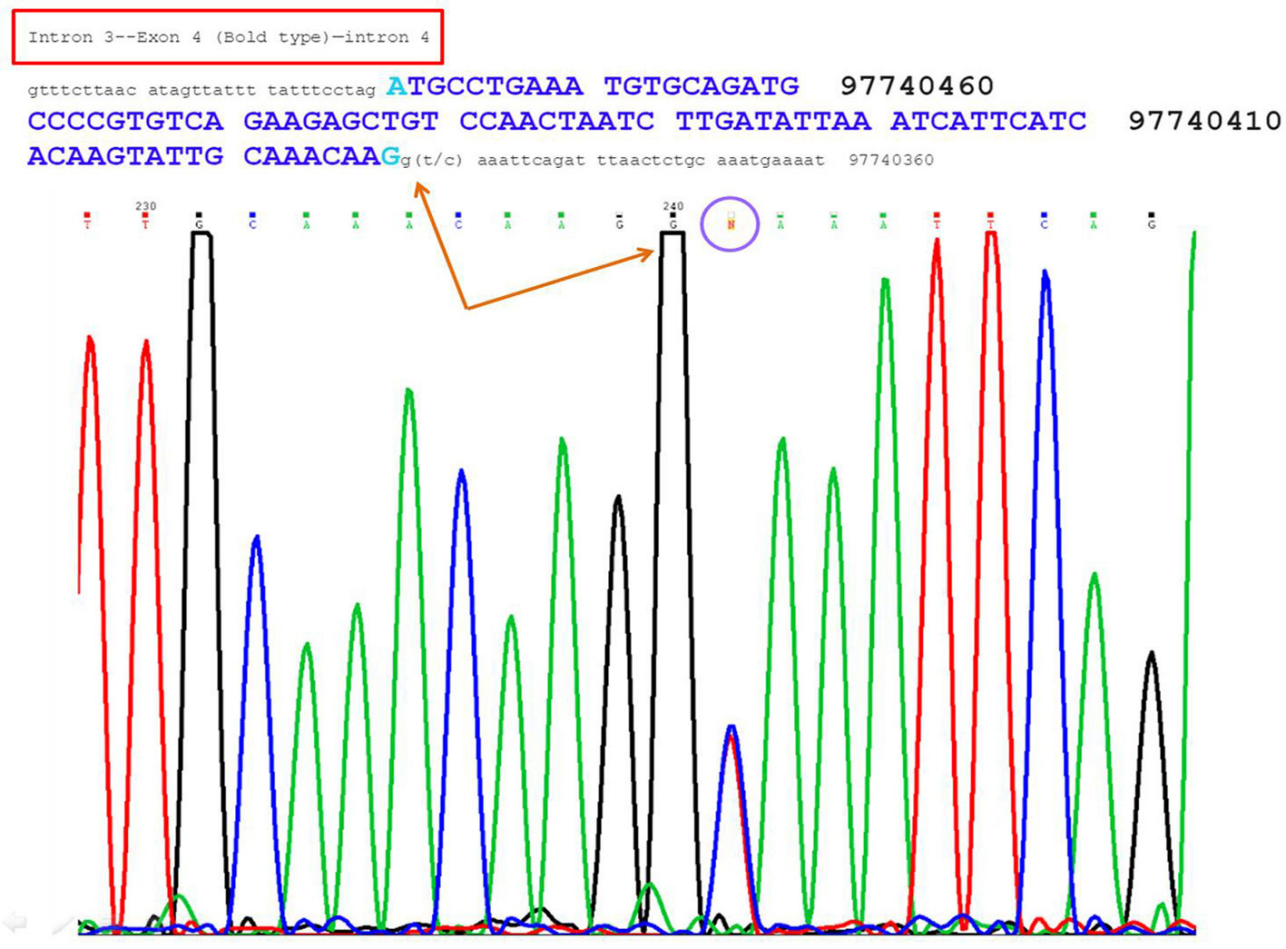
The figure showed a heterozygous site at the second base of the donor splice site of intron 4, i.e., G(T/C) AAATTCAGA.

**Figure 2:**
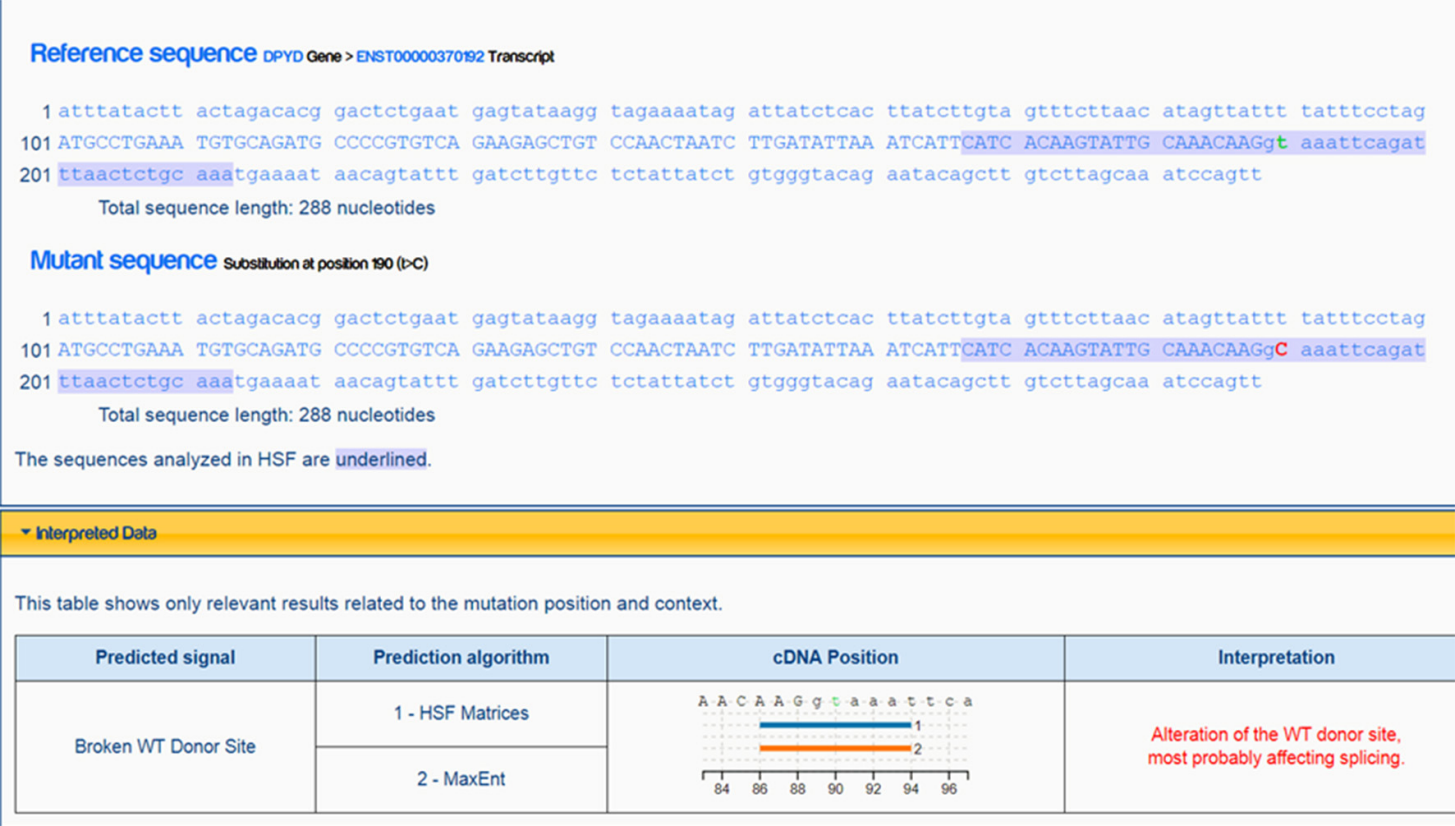
Analysis and prediction by Human Splicing Finder showing the mutation abolish the donor splice site of intron 4 of the DPYD gene resulting in exon skipping.

Exome sequencing confirmed that she is heterozygous for NM_000110.3: c.321+2T>C of the intron 4 of the ***DPYD*** gene. This mutation is not present in a population database, i.e., ExAC database (5). The variant NM_000110.3: c.321+2T>C affects the second nucleotide of the donor splice site of intron 4 of the ***DPYD*** gene (Figure 1). Bioinformatics analysis using Human Splicing Finder^1^ predicted that the NM_000110.3: c.321+2T>C abolish the donor splice site of intron 4 of the ***DPYD*** gene resulting in exon skipping (Figure 2). This mutation is predicted to be a non-functional allele of the DPYD gene.

Clinical Pharmacogenetics Implementation Consortium (CPIC) is an international consortium providing guidelines based on translating pharmacogenetic test result into an actionable prescribing decision for affected drugs. This can guide clinician on interpreting available genetic test results and ultimately ensuring a safer and drug delivery to patient (6).

Our reported patient had a heterozygous genotype and was considered having decreased DPD activity. The recommendation is to start with at least a 50% reduction of dosage upon resuming drug, followed by titration of dose base on tolerance and side effect profiles, aim to minimize toxicity while maintaining efficacy. That is, to increase the subsequent dose if she tolerated that dosage versus decrease the subsequent dose if there is still significant toxicity.

In the 2016 statement from FDA (US), there was insufficient data to recommend a specific dose in the patient with partial DPD activity for capecitabine (7). We decided to switch the subsequent remaining chemotherapy regimen to FOLFOX (every 2 week) for 11 cycles (oxaliplatin 85 mg/m2 at day 1, leucovorin 200 mg/m2 at day 1 and 2, 5-FU iv bolus (400 mg /m2) at day 1 and 2, 5-FU (600 mg/m2) iv infusion at 22 h at day 1 and 2.

We started with 30% dose 5-FU at cycle one FOLFOX in early January 2017. As patient tolerated the chemotherapy, we then stepped up the dosage of 5-FU to 40 and 50% dose at cycle II & III FOLFOX respectively.

However, after cycle III FOLFOX, she developed persistent grade 2–3 neutropenia for 2 weeks: neutrophil count around 0.8–1.2 (normal range 2.01–7.42), hence cycle IV FOLFOX was suspended for 3 weeks. We then stepped down the 5-FU dose back to 40% from cycle IV FOLFOX. The patient tolerated the subsequent cycles of chemotherapy and completed the whole course chemotherapy by early July 2017.

Supplementary Table 1 summarizes the events and progress in chronological order.

## Discussion

In Hong Kong, colorectal cancer has superseded lung cancer to claim the top spot in the overall ranking of new case incidence since 2013. It is also the second leading cause of cancer-related deaths in our locality^2^.

Despite advancement/discovery of novel chemotherapeutic agents, the fluoropyrimidine anti-cancer drug, especially the (5-FU) and its prodrug capecitabine still being the backbone of chemotherapy in adjuvant as well as palliative settings for colorectal cancer^3^.

DPD is the crucial initial enzyme for 5-FU catabolism and responsible for around 80% of degradation of 5-FU to the inactive metabolite. It has been estimated that about 3–5% of Caucasians have partial DPD deficiency and 0.2% have complete DPD deficiency (8).

When a standard dosage of fluoropyrimidines being prescribed to complete or partially DPD deficient patients, the decreased inactivation of 5-FU will result in excessive active anabolic products of 5-FU, leading to an increased risk of severe and even fatal toxicity (8, 9).

In general, the testing approach for DPD deficiency can be genotyping based or functional measurement/phenotype of DPD. Supplementary Table 2 summarizes the advantages and limitations of tests with these two approaches (10, 11).

Genetic polymorphism in ***DPYD*** is the leading cause of DPD deficiency. The ***DPYD*** gene is highly polymorphic with hundreds of mutations described so far, a few variants with genotype-guided dosing adjustment have been clinically validated, such as c.1905+1G>A (DPYD^*^2A), c.2846A>T, c.1679T>G, and c.1236G>A (12).

Besides, new deleterious ***DPYD*** variants in the noncoding gene region have been found upon thorough sequencing of the ***DPYD***. One of these includes a deep intronic variant in intron 10 (c.1129-5923C>G) mutation, resulting in a cryptic splice donor site, causing a shift in the reading frame and result in a premature stop codon and a truncated protein. Given the high prevalence of this mutation observed in European (2.6 % in Dutch and 3.3% in German population respectively), the authors concluded that screening for DPD deficiency should include a search for genomic rearrangements and aberrant splicing (13).

Pre-emptive screening of DPD deficiency before administrating fluoropyrimidine-based chemotherapy has been a standard practice in some European Countries: France (14), Italy and Netherlands ^4^. Majority of our current knowledge on ***DPYD*** is derived from studies on Caucasian population. Research on other ethnicities is crucial as a meta-analyses study revealed ethnical difference also has a role in genetic polymorphism in ***DPYD*** (15).

In the era of precision medicine, pharmacogenomics changes the landscape of cancer treatment and rewrite the natural history and outcome of many cancer patients/survivors.

There has been debate over the issue of cost-effectiveness on the pre-emptive screening of DPD deficiency before drug administration but recent studies achieved positive evaluation (16, 17). With the advent of Next Generation Sequencing technology, entire genome sequencing with affordable cost with a short turnaround time also enhance the feasibility of pre-emptive genotyping (18, 19).

Current evidence supports upfront ***DPYD*** screening for four ***DPYD*** variants (DPYD^*^2A, c.2846A>T, c.1679T>G, and c.1236G>A), followed by genotype-guided dosing, based on dosing recommendations by the CPIC. This clinical utility has been confirmed by two recent large-scale studies (20, 21) and drug label update has been called (22).

In conclusion, with a pre-emptive test on DPYD before the administration of the fluoropyrimidine drugs, the life-threatening event in this reported case could be avoided.

## Ethics statement

The study was jointly conducted at Department of Clinical Oncology, Queen Mary Hospital, Hong Kong, as well as Department of Clinical Oncology and Department of Pathology, The University of Hong Kong. The study was carried out in accordance with Declaration of Helsinki. Informed consent was obtained from the index patient and the study was approved by the Institute Review Board of Queen Mary Hospital.

## Author contributions

CT participated in the study's design and coordination, performed acquisition of data and drafted the manuscript. CL participated in exome sequencing test, data interpretation and revised the manuscript. KL and VL participated in study's design and revised the manuscript. M-YL revised the manuscript critically for important intellectual content. All authors read and approved the final manuscript.

### Conflict of interest statement

The authors declare that the research was conducted in the absence of any commercial or financial relationships that could be construed as a potential conflict of interest.

## Footnotes

^1^Available online at: http://www.umd.be/HSF3/ [Accessed 22 June, 2018].

^2^Website of Hong Kong Cancer Registry, Hospital Authority: http://www3.ha.org.hk/cancereg [Accessed June, 2018]

^3^NCCN guidelines: www.nccn.org

^4^Available online at: www.kennisbank.knmp.nl
