# Supplementary material for: A Novel DPYD Variant Associated With Severe Toxicity of Fluoropyrimidines: Role of Pre-emptive DPYD Genotype Screening
Source: Front Oncol. 2018 Jul 24;8:279. doi: 10.3389/fonc.2018.00279 (PMC6066555; doi:10.3389/fonc.2018.00279)
Supplement: Supplementary file 1 [file Table_1.DOCX]

| **Date** | **Event / Progress** |
| --- | --- |
| 3^rd^ September 2016 | Laparoscopic left hemi-colectomy done for carcinoma  of sigmoid colon  Pathologically stage T3N1M0 / III disease |
| 24^th^ October 2016 | CAPEOX (cycle one) started |
| 5^th^ November 2016  (Day 13) | Admitted for fever, diarrhea (grade 3)  Marrow suppression toxicity (grade 4):   - white blood cell: 0.52 x 10^9/L   (normal range: 3.89- 9.93 x 10^9/L) ;   - neutrophil: 0.04 x 10^9/L   (normal range: 2.01 – 7.42 x 10^9/L);   - platelet: 25 x 10^9/L   (normal range: 154 – 371 x 10^9/L)   - On IV piperacillin/tazobactam, growth factors |
| 29^th^ November 2016 | Discharged home |
| 20^th^ December 2016 | Exome sequencing result: novel DPYD variant present |
| 4^th^ January 2017 | cycle I FOLFOX with 30% 5-FU dose |
| 18^th^ January 2017 | cycle II FOLFOX with 40% 5-FU dose |
| 1^st^ February 2017 | cycle III FOLFOX with 50% 5-FU dose |
| 16^th^ February 2017 -  8^th^ March 2017 | Persistent grade 2-3 neutropenia:  Neutrophil: 0.8 -1.2 x 10^9/L  (normal range: 2.01- 7.42 x 10^9/L) |
| 15^th^ March 2017 -  5^th^ July 2017 | Cycle (IV – XI) FOLFOX with 40% 5-FU dose |

Table 1: summary of events and progress in chronological order
