# Supplementary material for: A Novel DPYD Variant Associated With Severe Toxicity of Fluoropyrimidines: Role of Pre-emptive DPYD Genotype Screening
Source: Front Oncol. 2018 Jul 24;8:279. doi: 10.3389/fonc.2018.00279 (PMC6066555; doi:10.3389/fonc.2018.00279)
Supplement: Supplementary file 2 [file Table_2.DOCX]

|  | Genotype of ***DPYD*** | DPD phenotype /  Functional measurement |
| --- | --- | --- |
| Methods/ approach | Comprehensive sequencing of germline DNA for deleterious mutations in ***DPYD***   - Next Generation Sequencing | - DPD activity in PBMCs - 2-[^13^C]-uracil breath test - Uracil/ dihydrouracil ratio - [^14^C]-thymine to dihydrothymine plasma ratio |
| Advantages | - High Specificity - unambiguous result - Not influenced by environment factors - Ready availability and implementation in many laboratories | - High sensitivity |
| Limitations | - Low sensitivity - Clinical validity is needed for novel or rare DPYD variants | - Affected by environmental external factors - Time-consuming - Materials/ equipment not readily available in laboratory |

**Table 2: Advantages and Limitations of tests with Genotype and Functional / phenotype based approaches** [13,14]

DPD = Dihydropyrimidine dehydrogenase

***DYPD*** = Dihydropyrimidine dehydrogenase gene

PBMCs = peripheral blood mononuclear cells.
